# Mineral and bone disorder after kidney transplantation (KTx)

**DOI:** 10.1590/2175-8239-JBN-2021-S113

**Published:** 2021-12-03

**Authors:** Carolina Lara Neves, Igor Dernizate B. Marques, Melani Ribeiro Custódio

**Affiliations:** 1 Universidade Federal da Bahia, Hospital das Clínicas, Salvador, BA, Brazil.; 2 Hospital Ana Nery, Salvador, BA, Brazil.; 3 Universidade Federal do Piauí, University Hospital, Teresina, PI, Brazil.; 4 Universidade de São Paulo, SP, Brazil.

## 1. Assessment frequency of biochemical and hormonal abnormalities

1.1 In the pre-KTx assessment, it is recommended to evaluate the mineral and bone profile [calcium (Ca), phosphorus (P), alkaline phosphatase (AP), parathormone (PTH) and 25-hydroxyvitamin (VitD)], and in the first 3 months post-KTx, serum calcium and phosphorus levels should be monitored weekly, or less frequently, according to clinical evolution (Opinion).

1.2 Within 3-12 months after KTx, the assessment frequency of laboratory tests will depend on the magnitude of biochemical changes and the established therapies (Opinion).

1.3 In the period over 12 months after KTx, the assessment frequency will depend on the function of renal graft (follow recommendations for CKD stages) and on the stabilization of biochemical changes, previously detected (Opinion).

## 2. Treatment of biochemical and hormonal abnormalities

2.1 Treatment of hyperparathyroidism after kidney transplantation or persistent HPT (pHPT) should take serum Ca levels into consideration (Evidence).

2.1.1 In the presence of mild or moderate hypercalcemia (tCa < 14 mg/dL or iCa < 1.80 mmol/L), treatment should be with cinacalcet or parathyroidectomy (PTX) if clinical treatment fails (Evidence). 

2.1.2 In the presence of severe hypercalcemia (tCa > 14 mg/dL or iCa > 1.80 mmol/L), treatment should be by PTX and, until its completion, with cinacalcet and/or antiresorptive agents (Evidence).

2.1.3 In the presence of normocalcemia, treatment of pHPT should follow the same recommendations for patients with CKD G3a-5D (Opinion).

2.1.4 The type of PTX in the treatment of pHPT should preferably be subtotal PTX (Opinion). 

2.2 P supplementation is indicated for patients with severe, symptomatic hypophosphatemia (P < 1.5 mg/dL) (Evidence).

2.3 Vitamin D supplementation should follow the same recommendations for CKD patients (Opinion).

## 3. Diagnosis and treatment of post-KTx osteopenia/osteoporosis

3.1 Bone densitometry (DXA), associated or not with FRAX, is the method of choice for assessing bone mass and risk of fracture after KTx (Opinion).

3.2 Bone biopsy should be considered before starting treatment with antiresorptive agents, for patients with eGFR < 30 mL/min/1.73m^2^ (Opinion).

3.3 The choice of treatment should consider the presence of biochemical and hormonal abnormalities of CKD-MBD (Evidence).

3.4 For patients in the 1st year after KTx, with low bone mass and eGFR > 30 mL/min/1.73m^2^, treatment with vitamin D, calcitriol and/or antiresorptive agents should be considered (Evidence).

3.5 For patients with CKD G4T-G5T with low bone mass, the same treatment is suggested as for non-dialytic patients with CKD G4-G5.

3.6 For all post-KTx patients with low bone mass, physical activity and cessation of smoking and alcohol abuse should be recommended. 

## Rational

Mineral metabolism disorders (MBD) are prevalent in the first 3 months post-KTx, and some metabolic changes persist, such as elevated PTH levels and/or hypercalcemia, characterizing pHPT^1^ ^-^ ^4^. Hypophosphatemia is observed immediately after KTx in 50% of patients, caused by the reestablishment of the glomerular filtration function and by the elevated serum levels of PTH and FGF23, which lead to increased phosphaturia. Prolonged hypophosphatemia, serum P < 2.4 mg/dL, can lead to disturbances in bone mineralization. However, phosphorus replacement should be avoided, since it contributes to increase serum PTH levels, being recommended only in cases of severe and symptomatic hypophosphatemia (serum P < 1.5 mg/dL)^1^ ^,^ ^5^ ^,^ ^6^.

The presence of hypercalcemia ranges from 11% to 31%, with some studies showing an incidence of up to 50%, depending on the severity of SHPT at the time of KTx and dialysis vintage^1^ ^,^ ^2^ ^,^ ^3^. The main etiology of hypercalcemia is pHPT, since, with the return of renal function, there is an improvement in bone resistance to PTH action, increased calcitriol synthesis, increased intestinal Ca absorption and distal tubular Ca reabsorption^4^ ^,^ ^7^. In addition, other factors contribute to the presence of hypercalcemia, such as resorption of vascular and ectopic calcifications, prolonged postoperative immobility, and abrupt discontinuation of post-KTx cinacalcet, which, associated with high PTH levels, can lead to severe hypercalcemia. In the late period of KTx, episodes of hypercalcemia should be investigated to exclude neoplasms or serious systemic infections. Hypercalcemia-associated complications are the presence of tubulointerstitial calcifications (nephrocalcinosis), association with chronic graft nephropathy, and exacerbation of aortic calcification^7^ ^,^ ^8^ ^,^ ^9^. 

Treatment of mild to moderate hypercalcemia (iCa 1.40 to 1.80 mmol/L) includes suspension of Ca supplements, use of thiazides and, frequently, use of cinacalcet, provided there is a satisfactory response to low doses of the medication (30-60 mg); otherwise, subtotal PTX is recommended. The treatment of severe hypercalcemia (iCa > 1.80 mmol/L), in addition to the aforementioned measures, recommends intravenous hydration with crystalloid solutions, use of loop diuretics, short-acting bisphosphonates such as pamidronate 60-90 mg/dose every 1-3 months and/or cinacalcet while awaiting PTX. 

Serum PTH levels fall rapidly between 3-6 months and stabilize at 6-12 months after KTx and, to establish whether they are normal or not, it is always necessary to draw a parallel with the current glomerular filtration rate. The prevalence of pHPT ranges from 25-80% among studies, depending on the serum PTH level considered in the normal range/renal function, since there is no agreement in literature on the optimal post-KTx PTH. Most authors consider a range of serum PTH levels between 100-150 pg/mL acceptable for patients with glomerular filtration ≥ 30 mL/min^1^ ^,^ ^10^ ^,^ ^11^ ^,^ ^12^.

The pHPT may be associated or not with hypercalcemia, as in cases of significant loss of graft function, equivalent to CKD in stages 3-5. Treatment should be started with cinacalcet and, in cases of failure or severe pHPT, subtotal PTX is indicated. 

There is a gap regarding randomized studies in post-transplant that show, in addition to calcemia and PTH control, improvement in bone mass, graft function, fracture reduction, as well as the use of cinacalcet *versus* PTX. 

A systematic review/meta-analysis published in 2012 reports that most studies, using cinacalcet for the treatment of pHPT and hypercalcemia, were not randomized, but showed good control of Ca and PTH^13^. Two clinical studies presenting small case series showed benefit from the use of cinacalcet in the control of hypercalcemia and improved bone mineral density of the radius and hip^14^ ^,^ ^15^. On the other hand, four studies comparing the use of cinacalcet versus PTX in patients with pHPT showed that patients undergoing PTX had better control of calcemia and PTH levels^16^ ^,^ ^17^ ^,^ ^18^ ^,^ ^19^.

Subtotal PTX seems to be the most effective and efficient treatment for hypercalcemia associated with pHPT. Although slight worsening of kidney function may occur after the procedure, it is usually transient and does not decrease graft survival^20,21^. Some authors suggest that, when possible, surgery should be indicated after the first year of KTx, since the risk of worsening kidney function is lower, although there is disagreement with respect to this schedule^22^ ^,^ ^23^. The pHPT is associated with worsening of graft function^11^ ^,^ ^24^, increased risk of fracture^25^ ^,^ ^26^ and mortality ^27^. Considering the complications caused by pHPT and hypercalcemia, it is recommended, when possible, to perform PTX before KTx^13^ ^,^ ^28^. 

Currently, the life expectancy of patients and kidney grafts has increased significantly. Thus, the prevention of complications is important, such as bone disease, which leads to diffuse pain, fractures, deformities, and limitations. It is known that, even after successful KTx, in addition to pHPT or other bone disease, the patient may also present with osteopenia/osteoporosis developed before or after transplantation. The association of these metabolic bone changes increases the risk of fractures, favors the early onset of hypercalcemia and hypophosphatemia and, later, acute rejection and mortality^12^. For this reason, as stated earlier, it is recommended that control of the patients' bone disease occurs prior to KTx, either with medication or subtotal PTX.

Bone mass loss prior to KTx (verified by DXA) occurs especially in patients in which the etiology of CKD requires prolonged corticosteroids use. After KTx, bone loss occurs more in the lumbar spine, due to the action of immunosuppressants, but increasingly less significant with the new regimens^29^ ^,^ ^30^. However, risk factors persist, such as those observed in the general population: age, female gender, sedentary lifestyle, inadequate nutritional status, chronic use of corticosteroids, previous fracture, and diabetes *mellitus*. 

FRAX is a tool that is used in association with DXA to assess risks of fracture. Although not specific for CKD patients, FRAX has been recognized and validated for this population as well, providing information on the 10-year risk of hip fracture^31^. Another test that complements the data provided by DXA (quantity of bone mass) is the high-resolution peripheral quantitative computed tomography (HR-pQCT), which assesses the quality of bone tissue^32^. Unfortunately, the performance of HR-pQCT is restricted to a few diagnostic centers and it is not routinely used.

In clinical practice, the suggested practice for treatment of osteoporosis in patients after KTx should be based on the control of existing metabolic changes and on the institution of general measures, such as changing habits and lifestyle, introduction of physical exercise, smoking cessation, moderation in alcohol consumption, among others^33^. These measures aim to stimulate increased bone mass and improve balance, preventing falls and fractures, and thus improving quality of life. Drug treatment for osteoporosis should be individualized. In the general population, this treatment is well established, with several drugs available that could reduce bone mass loss and/or stimulate bone mass formation, reducing the incidence of fractures^33^. However, the use of these drugs in patients after KTx, with glomerular filtration above 30 mL/min, presents some particularities:

## Vitamin D

The incidence of hypovitaminosis D in patients after KTx is around 50%. Vitamin D replacement is important for reducing bone mass loss, but it is contraindicated in the presence of hypercalcemia ^33^.

## Bisphosphonates 

Bisphosphonates are widely used, due to their effectiveness and low cost, in patients in the general population and in transplant patients. The widespread and preventive use of bisphosphonates, in the loss of bone mass immediately after KTx, has been questioned even with studies showing that this medication preserves bone mass without interfering with PTH levels^34,^ ^35^. However, as mentioned above, bone mass loss in the central skeleton no longer occurs as significantly as before^36^ due to new immunosuppressive regimens, low rates of acute rejection, decreased use of glucocorticoids, and widespread use of vitamin D. Furthermore, there are studies showing that the use of this medication does not reduce the risk of fracture in this population^36^ ^,^ ^37^. 

Bisphosphonates inhibit osteoclast function, and without careful monitoring, may cause a decoupling between bone formation and resorption, inducing the development of low turnover (adynamic) bone disease or mineralization defect. Although serum PTH levels and other bone markers do not reflect bone histology, patients with suspected or diagnosed adynamic bone disease should not receive bisphosphonates. Recently, some studies have shown that bisphosphonates do not induce adynamic bone disease, as demonstrated in previous publications^38^ ^,^ ^39^ ^,^ ^40^, but their indication remains controversial in KTx patients with glomerular filtration lower than 30 mL/min/1.73m², similar to patients in CKD stages 4, 5 and 5D^1^.

A study by Marques et al., using HR-pQCT and bone biopsy, demonstrated that KTx induces a loss of bone tissue connectivity, especially in the peripheral skeleton, where most fractures occur^41^. This fact justifies the fracture in patients with DXA within the normal range, demonstrating that bone changes occur in its microarchitecture. Therefore, bisphosphonates should be considered for patients at high risk of fracture, evidenced by loss of bone mass, especially at these sites.

## Denosumab

Denosumab increases bone mass mainly in the lumbar spine, but it also has a positive impact on the femoral neck. This difference observed between the two sites is justified by the greater action of denosumab on trabecular bone, which is more prevalent in the lumbar spine. A study comparing the efficacy of denosumab and bisphosphonates showed that bone mass increased in the lumbar spine and femoral neck in both arms of the study, with this increase being more important in the denosumab group^42^.

Mechanisms that justify these differences:

1. Inhibitory effect: bisphosphonates are absorbed by the mature osteoclast, and thus inhibit the resorptive action of this cell. On the other hand, the action of denosumab is more effective in reducing bone resorption, by preventing osteoclasts maturation, activation and survival. 

2. Antifracture efficacy: denosumab has a greater impact on cortical bone, since, in addition to acting on bone mass, it improves microarchitecture parameters, as evidenced by a study using HR-pQCT^43^. By its action, altering bone microarchitecture, denosumab promotes a more complete inhibition of bone resorption and reduces the risk of fracture when compared to bisphosphonates.

The side effect of denosumab is hypocalcemia, which may occur even with stable PTH levels and may be prevented and/or corrected with concomitant use of calcitriol^44^ ^,^ ^45^. Despite this, it is a safe and effective drug in the treatment of osteoporosis in kidney transplant patients, and could be used at any stage of graft dysfunction.

## Other drug therapies

The use of recombinant human parathyroid hormone, teriparatide, has no consistent data for the treatment of post-KTx osteoporosis. Cejka et al. showed that after 6 months of teriparatide use there was no improvement in bone mass, histology or bone turnover markers compared to control group ^46^. It is probably indicated in some cases of persistent hypoparathyroidism that may occur in transplant patients undergoing PTX.

Regarding hormone replacement in transplant patients, there are no consistent studies that indicate the best therapy in terms of efficacy, safety and doses of the drugs already available. Early menopause occurs in women at all CKD stages, and clinical trials are needed to define the best therapy and the impact of CKD on menopause ^47^. 
